# Mental Well-Being (Depression, Loneliness, Insomnia, Daily Life Fatigue) during COVID-19 Related Home-Confinement—A Study from Poland

**DOI:** 10.3390/ijerph17207417

**Published:** 2020-10-12

**Authors:** Adrian Bartoszek, Dariusz Walkowiak, Agnieszka Bartoszek, Grzegorz Kardas

**Affiliations:** 1 Faculty of Medicine, Medical University of Lodz, 90-001 Lodz, Poland; 2 Department of Pathophysiology, Medical University of Lublin, 20-090 Lublin, Poland; 3 Department of Organization and Management in Health Care Poznan University of Medical Sciences, 60-356 Poznań, Poland; dariuszwalkowiak@ump.edu.pl; 4 Department of Family Medicine and Community Nursing, Medical University of Lublin, 20-081 Lublin, Poland; agabartoszek@wp.pl; 5 Clinic of Internal Medicine, Asthma and Allergy, Medical University of Lodz, 90-001 Lodz, Poland; kardas7@gmail.com

**Keywords:** COVID-19, depression, loneliness, insomnia, fatigue, home confinement/isolation

## Abstract

The COVID-19 pandemic is a great threat to both physical and mental health as it may lead to psychological stress connected with an economic crisis, threat of unemployment, or fear of losing family members. Emerging data shows that the general public may be vulnerable to the pandemic-related stress and experience frequently prevalent anxiety. A study involving 471 subjects (85.6% female) was conducted online during the COVID-19 pandemic. We used the following scales: Insomnia Severity Index (ISI), Beck Depression Inventory (BDI), Revised University of California, Los Angeles (R-UCLA) Loneliness Scale, and Daily Life Fatigue scale (DLF). Women had higher mean scores of depression, loneliness, and daily life fatigue and more often than males started exercising. Among people professionally active before the pandemic, there were more cases of increased alcohol consumption than among students. No differences in alcohol consumption patterns were found between genders. People living alone had higher scores of loneliness and daily life fatigue compared to those living with someone. Respondents who started taking any new drugs during COVID-19 home confinement had higher outcomes in all questionnaires. During home confinement, high scores of depression, insomnia, loneliness, and everyday fatigue were observed.

## 1. Introduction

The outbreak of the global COVID-19 pandemic revealed that the world had been completely unprepared for it. As of 25 May 2020, the global number of reported cases has reached 5.2 million and is still rising [1]. So far, there is no specific anti-COVID-19 treatment, nor targeted prevention. Fortunately, currently over 50 vaccine candidates are in pre-clinical studies with some in Phase I [2]. Hopefully, an effective vaccine is expected to be available in early 2021.

The first confirmed COVID-19 case in Poland was announced on 4 March 2020. On 24 March, the Polish government announced strict restrictions, which forced the vast majority of Polish citizens to obey home confinement. This meant that people were obliged to spend most of their day at home—schools, universities, public institutions operated remotely (online), most of the workers were asked to work from home. The restrictions included: mandatory covering of mouth and nose in public spaces, keeping a minimum two-meter distance between pedestrians, the cancellation of public events, limited number of customers in shops, closed shopping centers, international travel ban, and closed airports. On 20 April the Polish government started to gradually abolish the restrictions.

The global health crisis of the COVID-19 pandemic is a great threat not only in terms of physical health. As multiple countries worldwide have decided to apply the technique of social distancing, this creates a huge challenge in terms of mental health, greatly reinforced by other factors that may lead to psychological and psychiatric disturbances, such as economic crisis, the threat of unemployment, or fear of losing family members. Emerging data show that the general public may be vulnerable to pandemic-related stress and frequently experience prevalent anxiety [3]. Psychological and psychiatric disorders are complex in terms of pathogenesis with multiple causative factors including genetics, physical state, socioeconomic status, etc. However, in general, psychological and social factors may play a role in triggering some of these disorders (e.g., anxiety or depression). In light of that, social distancing and home confinement regulations are important factors that shall be considered in managing social and psychological well-being [4,5,6,7,8].

Since the beginning of the COVID-19 pandemic at the end of 2019, some research on mental health has been published. The issues covered include mostly depression and anxiety, with some preliminary reports also on insomnia. However, these reports are mostly from China and other Asian countries, where the pandemic first started and lessons on the issue were learned first [9,10,11]. Some reports from other regions confirm a declining tendency in peoples well-being and mental condition during the COVID-19 pandemic [12,13,14]. Moreover, many of these studies are focused on the mental health of healthcare workers [15], who are on the frontline of the pandemic, yet fewer studies are focused on those not involved in healthcare [16]. To date, very limited data is available on the issue of the impact of the COVID-19 pandemic on mental health in European Countries [17]. Only one study covered some aspects of this topic in Poland, whose authors studied the effects of mandatory face mask restrictions on anxiety, depression, insomnia, and social dysfunction questionnaire scores [18].

As described above, certain areas of research on mental health during home confinement were not covered, particularly, especially regarding the geographic distribution of the studies. Moreover, in June 2020 a position paper was published in The Lancet with a call-for-action for studies on mental health effects of the COVID-19 pandemic across population and vulnerable groups [19]. This is particularly important as not only is the pandemic still active, but the world may also face its second wave. In fact, some countries already re-implemented social distancing requirements and home confinement regulations.

To extend the scope of the research on the topic, we decided to include some further factors that may be associated with varying mental health outcomes during COVID-19 home confinement. These factors included: alcohol consumption, taking psychiatric drugs (antidepressants, anxiolytics, sedatives or others), and living alone or with others during home confinement.

The aim of the study was to measure indicators of mental well-being in a Polish sample with regard to selected sociodemographic and health behavior data during home confinement related to COVID-19 pandemic. We suspected that selected sociodemographic and health behavior data could differentiate mental well-being during home confinement related to COVID-19 pandemic.

## 2. Materials and Methods 

### 2.1. Study Design and Participants

This was an online study involving Polish adult citizens, conducted during the COVID-19 pandemic. Current/recovered COVID-19 patients were excluded from participation in the study. Study participants were invited to complete a self-administered online questionnaire (Google Forms) 1.5 weeks after home confinement restrictions were introduced in Poland (3 April 2020) and the questionnaire was available for the following two weeks. Participation in the study was generally requested through the researchers’ university emails and their official websites. A request for participation was also sent via the researchers’ institution social media profile (Facebook).

### 2.2. Measurement Tools 

The survey was created on the basis of a literature review on mental well-being and with the use of standardized research tools examining symptoms of depression, loneliness, insomnia, and fatigue. Sociodemographic questions concerning age, gender, professional status, number of household members, and the questions related to alcohol consumption, physical activity, and administration of new drugs at the time of questionnaire filling (that is during current COVID-19-related home confinement) were collected. We also asked about the date at which an individual began his/her home confinement.

The Insomnia Severity Index (ISI) was used to measure the potential severity of insomnia. Each item of that tool is rated on a 0–4 scale, and its total score ranges from 0 to 28. A higher score suggests more severe insomnia symptoms [20]. The scale is widely used in Polish population [21]. Internal consistency of ISI, assessed with Cronbach’s α and McDonald’s ω, was found to be sufficient for the study (α = 0.898, ω = 0.901).

The Beck Depression Inventory (BDI), developed by Aaron T. Beck, is a self-administered questionnaire consisting of 21 multiple-choice questions. It is one of the most commonly used instruments for measuring the severity of depression. The global score of BDI is an arithmetic summation of the ratings of all 21 symptoms scored on a final scale ranging from 0 to 63. The higher the global score, the greater the depression level [22]. The scale has been validated in Poland [23]. Internal consistency of BDI was found to be sufficient for the study (α = 0.904, ω = 0.909).

Revised University of California, Los Angeles (R-UCLA) Loneliness Scale is a 20-item scale designed to measure one’s subjective feelings of loneliness as well as feelings of social isolation. Participants rate each item on a scale from 1 (Never) to 4 (Often) [24]. The scale has been validated to Polish conditions [25]. Internal consistency of R-UCLA was found to be sufficient for the study (α = 0.791, ω = 0.801).

The Daily Life Fatigue scale (DLF) is a Polish questionnaire which consists of three subscales: physical fatigue (PF), associated with daily physical activity; mental fatigue (MF), associated with the mental activity (e.g., calculations in memory or remembering a phone number); and social fatigue (SF), regarding social activities, e.g., planning time with loved ones. The result of the scale is a measure of general everyday fatigue, which is the sum of points obtained in all 24 positions of the three subscales in the range from 0 to 24. The higher the score, the higher the level of general everyday fatigue felt [26]. Internal consistency of DLF was found to be sufficient for the study (α = 0.826, ω = 0.829).

### 2.3. Data Collection

Participation in the study was fully voluntary. All participants were familiarized with the study conditions and gave informed consent to participate. Confidentiality and anonymity were maintained as no data that potentially identified a responder was collected. Bioethics Committee confirmed that according to Polish law and Good Clinical Practice regulations this research does not require an approval of a Bioethics Committee (KB nr 542/20).

### 2.4. Statistical Analysis

The data collected in the questionnaires were verified and checked for completeness, quality, and consistency. Then it was coded and exported into statistical packages: JASP (Version 0.12.2; University of Amsterdam, Amsterdam, The Netherlands) and STATISTICA 13.1 (TIBCO, Palo Alto, Santa Clara, CA, USA). For all scales, the mean, median, and standard deviation (SD) values were calculated. Welch’s unequal variances *t*-test was used to compare differences between sub-groups (gender, age, activities, and others). Effect size is given by Cohen’s d. We used Kendall’s Tau b to measure the strength of the relationships between variables. A 5% level of significance was used for all hypothesis tests.

## 3. Results

### 3.1. Group Characteristics

Sociodemographic characteristics and other personal data are presented in Table 1. Generally, most of the respondents were women, students. Standard deviation (SD)

### 3.2. General Scores of Mental Well-Being during COVID-19 Related Home-Confinement

The average DLF result was 7.541, with results ranging from 0 to 24 (Table 2). The results of BDI ranged from 0 to 54, with a mean score of 14.161. The average R-UCLA result was 42.041, ranging from 20 to 74. The mean ISI result was 15.781, with results in a range of 7–35. In our group, 86% participants scored above the cut-off point of ISI questionnaire for acute/chronic insomnia (>7 points). The average isolation period of our respondents was 20 days (range 3.5–24). We did not observe the impact of isolation time on BDI, R-UCLA, and ISI results. For DLF, an effect of isolation time on results was observed (Kendall’s Tau b = 0.064, *p* = 0.043).

### 3.3. Gender Differences for the Mental Well-Being during COVID-19 Related Home-Confinement

The results of all scales were analyzed in terms of age, activities, solitude, employee vs. student status, traditional work vs. telework, and gender. Statistically significant differences in the reaction to the new social experience of isolation and social distancing were only observed between genders (Table 3). Women had significantly higher mean scores in DLF, BDI, and R-UCLA, but not in ISI, where the difference was borderline, but higher than the assumed level of statistical significance.

### 3.4. Gender Differences in Physical Activity during and Occupation Disparities for Alcohol Consumption during COVID-19 Related Home-Confinement

Among the other factors examined in connection with the social isolation period were also issues related to physical activity. While individual groups (female /male, working/not working) did not differ in the declared frequency of leaving the house, it turned out that there were some differences regarding lifestyle choices. No statically significant differences in physical activity among genders was shown (see Table 4A). In the group of people professionally active before the pandemic, there were more cases of drinking alcohol more or more often than in the case of students (Table 4B). No differences in alcohol consumption patterns, however, were found between genders.

### 3.5. Impact of Living Alone, New Drug Administration, and Declared Alcohol Consumption on the Mental Well-Being during COVID-19 Related Home-Confinement

We also studied the impact of living alone on the well-being of people during pandemic-enforced isolation. Table 5A presents the results of the scales broken down into two groups, one where the respondents were in home confinement with a partner or family and the second group in which people were living alone. Differences for individual scales between people who started taking new drugs during a pandemic and those who did not were also investigated (Table 5B). As for depression, measured with BDI, the mean score in the group that declared increased alcohol consumption was 17.82 compared to 13.67 in the group that declared not consuming more alcohol (BDI cut-off for mild depression is >13).

## 4. Discussion

Research on mental health during the COVID-19 pandemic is an important study area that possibly concerns many individuals, considering that the government announced strict restrictions, which forced the vast majority of Polish citizens to obey home confinement, to study and work remotely, and to adjust to the new situation. For this reason, our study aimed to measure mental well-being (levels of depression, insomnia, daily life fatigue, and loneliness) in a sample of the Polish population during COVID-19 related home confinement. We focused on differences between genders, physical activity, alcohol consumption, drugs, and living alone.

In a review of studies on depression among healthcare workers (over 33,000 cases) the depression prevalence was estimated to be 23%. Moreover, another review on depression, which was focused on the general population, showed a 33.7% depression prevalence. This clearly shows the scale of psychological and psychiatric problems related to the pandemic, both among healthcare workers—who in their previous work have already dealt with numerous stressful situations related to other peoples’ health—and the general public, for whom this situation may be a kind of a reminder of value and fragility of humans’ health [10,27].

A very recent study from China, where the authors used similar methodology as we did in studying insomnia (online ISI questionnaire, pre- and during the pandemic) found a significant increase of insomnia (ISI > 7) from 26.2% to 33.7%. The authors of that study conclude that their findings suggest that insomnia is associated with the COVID-19 outbreak-related psychological reactions and poor sleep hygiene [28].

Another recent report from Greece found that 37% of respondents scored above the cut-off score for insomnia. In our study, this percentage was 86%. Both of these values are above the generally reported worldwide insomnia (acute and chronic) prevalence, estimated before the pandemic to be between 3.9% and 22% [29]. These findings may be an indication that some exacerbations of sleep disturbances happen during the COVID-19 pandemic. Moreover, differences in insomnia scores between genders were confirmed in the cited study from Greece, contrary to our results [17]. Some explanation of these findings may be that during home confinement, people experience less physical fatigue and sun exposure, as well as possibly tend to more prominently use electronic devices that altogether may influence sleep homeostasis [30,31,32]. Also, without a doubt, the psychological stress caused by this unprecedented situation greatly contributes to this phenomenon.

A study similar to ours was carried out in Italy online on 2291 respondents during March and April 2020. The results have clearly shown that the pandemic and associated psychological stress are risk factors for sleep disorders and psychological diseases, e.g., the authors have shown that all of the elements of the Psychological Well-Being (PGWB) questionnaire (anxiety, depressed mood, positive well-being, self-control, general health, vitality) were significantly worse among study respondents than in previous general population data [33].

In a very recent meta-analysis of studies on depression, anxiety, and insomnia among healthcare workers during the COVID-19 pandemic the authors analyzed the results of 13 articles on these subjects, all of which were 2020 reports from Asia (China predominantly). Insomnia was found in five out of these 13 studies. The conclusion of this meta-analysis was that insomnia prevalence during the COVID-19 pandemic was 34.32%. The authors did not perform gender sub-group analysis due to limited data available [10].

Although the issue of insomnia is complicated and little is known on the impact of COVID-19-related stress on it, we may find some practical recommendations addressing this issue. In April 2020 the European cognitive behavioral therapy for insomnia (CBT-I) task force published their recommendations on dealing with sleep problems during this pandemic [34].

Existing reports show positive correlations between home confinement during COVID-19 pandemic and perceived loneliness. Tull et al. have shown this using the UCLA questionnaire in 500 U.S. adults. Surprisingly, in the same study, the perceived impact of COVID-19 itself was negatively associated with loneliness and additionally positively associated with social support [35]. Studies suggest that loneliness during COVID-19 home confinement is more strongly felt by younger people [36]. However, interventions addressing loneliness during COVID-19 home confinement, which are discussed in literature, are proposed to be particularly important among the elderly [37,38]. These include, for example, telehealth and online group interventions targeting these psychological needs [39]. Although societies have—in a way—become used to the fact that loneliness concerns the elderly, in these difficult times it has affected almost every age group. In fact, regardless of the age group, the possibility of psychological support and maintaining social life, e.g., online, should provide an opportunity to improve individual’s well-being.

A specific type of psychological fatigue is the Daily Life Fatigue (DLF). The author of the questionnaire (Urbańska) defines DLF as subjective overall fatigue, which is expressed by the reluctance to undertake daily physical, mental, and social activities [26]. Similar to the other scales used in the study, women had significantly higher mean scores in DLF, comparing to men. Living alone and taking new drugs during COVID-19 home confinement, were both variables associated with higher scores of DLF. Of importance in the perceived DLF is the self-interpretation of an individual’s situation; one may subjectively feel considerably tired while having objectively few everyday activities and vice versa.

To the best of the authors’ knowledge, there has not yet been a study that addressed the issue of DLF during COVID-19 home confinement. This may be caused by the fact that the DLF scale is a relatively novel tool, developed in Polish and has no English translation and validation yet. However, a study that covers the topic of ‘psychological fatigue’ in Turkey has recently been published by Morgul et al. The authors concluded that although knowledge, attitudes, and behavior concerning COVID-19 preventive measures are important to prevent transmission of the disease, they are also associated with participants’ fatigue. This, in turn, might lead to a psychological outcome e.g., pandemic-related fear and anxiety [40].

We found that, compared to pre-pandemic times, higher declared alcohol consumption is associated with higher Beck depression inventory scores. This outcome implies that some specific caution regarding addiction and mental health should be addressed to those people—both by healthcare professionals and, most importantly, by family members that are together during home confinement. This is particularly important as alcohol misuse during the COVID-19 pandemic may be considered one of the potential public health crises [41], since rising alcohol sales and consumption have been reported in European countries [42].

With social isolation and home confinement, there are also a number of social consequences related to mental health. Among them, it is worth mentioning the risk of intensification or exacerbation of domestic violence, which has been highlighted in the literature [43]. This and other themes, combined with the observations on mental health, should particularly lead to interventions aimed at improving people’s well-being.

## 5. Strengths and Limitations

This is one of the very first studies on the impact of COVID-19 pandemic on mental health in Europe. In particular, it is only the second covering this topic in Poland. To date of article submission, one report from Poland has shown that face mask restrictions, apart from preventing the spread of COVID-19, may in a way increase the level of perceived self-protection and social solidarity, which may improve the general mental health well-being [18].

Certain limitations apply to the results of our study. First of all, it is important to acknowledge this was an online only study which was performed via sharing the link to the survey. This implies that the results are limited to those with access to the Internet only. Moreover, we acknowledge that our study group is predominantly female, yet we believe that the statistical analysis we provided still allows an objective analysis of the results obtained. Moreover, gender differences in R-UCLA, BDI, and DLF have strong statistical significance. Due to the study design (social media) we are unable to provide the response rate to the survey.

With time and downturn of the epidemic in Poland, the home confinement regulations were (and at the time of article submission constantly are) being loosened, thus further data collection would possibly result in biased results.

The final limitation of our research is that it is a survey from which a nation-wide scale should not be directly extrapolated. This would have been possible only with nationally representative study. However, we believe that our results may still provide useful information on the impact of COVID-19 pandemic on the peoples’ mental health. We have shown some general tendencies across genders that imply that further studies in the area may be needed. The other issue is the 18–30 age predominance in our study group. However, we found that studies with a similar approach to ours (web-based questionnaire studies during COVID-19 in Europe) faced similar problems of female and 18–30 sample predominance [17]. Self-selection bias for interest in psychological themes next to bias due to the online administration system is possible.

What we report are results on depression, insomnia, loneliness, and fatigue across COVID-19 free individuals. However, it should be remembered that the above-mentioned problems also apply to patients diagnosed with COVID-19, as reported in the literature [44].

## 6. Conclusions

The outbreak of any infectious disease is associated with panic among people. People’s response to a pandemic determines not only the rate of the spread of the disease but also psychosocial disorders both during and after the pandemic. Despite this, there are not enough tools that could help maintain the social well-being of the population. This is understandable at the beginning of the spread of the disease where all funds are directed directly to fighting the disease.

As the pandemic progressed, severe restrictions were imposed in many countries to keep people at home. This can lead to further disorders related to social isolation. During homestay, people have higher scores of depression, insomnia, loneliness, and everyday fatigue. An important role in the development or deepening of mental disorders can be caused by new drugs that inadequately administrated can cause an increase in the number of hospitalizations, which is not desirable during a pandemic. Most mental health studies focus on healthcare workers, while the society at large is usually overlooked. We intended to pay special attention to these disorders that may appear in society during isolation because it is as important as fighting the disease itself, and the consequences can be serious in the long run.

## Figures and Tables

**Table 1:** Demographic and complementary data, *n* = 471.

Group Characteristics	*N* (%)
Gender	
female	403 (85.6)
male	68 (14.4)
Mean age (years)	25.5 (SD = 2.1) (range 18–74 years)
Occupation	
Student	290 (61.6)
Worker Student and Worker	132 (28) 49 (10.4)
Mean time of isolation	20 days (3.5–24)
Physical activity (compared to before isolation)	
No new exercising Started exercising during isolation Less exercising Same amount of exercising More exercising	148 (31.4) 76 (16.1) 86 (18.3) 87 (18.5) 74 (15.7)
Declared alcohol consumption (compared to before isolation)	
No alcohol consumption Same amount of alcohol consumed More alcohol consumed	233 (49.5) 182 (38.6) 56 (11.9)
Living alone during COVID-19 home confinement	
Yes No	57 (12.1) 414 (87.9)
Declared taking of any new drugs during COVID-19 home confinement	
Yes No	425 (90.2) 46 (9.8)

**Table 2:** Results of Daily Life Fatigue (DLF) scale (with Physical (PF), Mental (MF), and Social Fatigue (SF) subscales), Beck Depression Inventory (BDI), the Revised University of California, Los Angeles (R-UCLA) Loneliness Scale (R-UCLA), and Insomnia Severity Index (ISI) across study group, *n* = 471.

Parameter	DLF			DLF	BDI	R-UCLA	ISI
	PF	MF	SF				
Mean	2.28	2.88	2.38	7.54	14.16	42.04	15.78
Median	2.00	3.00	2.00	6.00	13.00	40.00	15.00
SD	2.22	2.47	2.17	5.91	10.64	11.78	6.95
Minimum	0.00	0.00	0.00	0.00	0.00	20.00	7.00
Maximum	8.00	8.00	8.00	24.00	54.00	74.00	35.00
25th percentile	0.00	1.00	1.00	2.00	5.00	32.25	10.00
75th percentile	4.00	5.00	4.00	12.00	21.00	50.75	21.00

**Table 3:** Gender differences for the Daily Life Fatigue scale (DLF), the Beck Depression Inventory (BDI), Revised UCLA Loneliness Scale (R-UCLA), and the Insomnia Severity Index (ISI) scales scores, *n* = 471. Confidence interval (CI).

		DLF		BDI		R-UCLA		ISI	
Gender		Female	Male	Female	Male	Female	Male	Female	Male
Valid		403	68	403	68	403	68	403	68
Mean		7.84	5.75	14.73	10.78	42.51	39.36	16.01	14.46
Median		7.00	5.00	13.00	7.50	41.00	37.00	15.00	13.50
SD		6.05	4.65	10.81	8.91	11.74	11.75	7.02	6.38
Minimum		0.00	0.00	0.00	0.00	21.00	20.00	7.00	7.00
Maximum		24.00	18.00	54.00	37.00	74.00	70.00	35.00	27.00
t		3.28		3.27		2.097		1.83	
df		109.24		103.34		97.27		96.51	
*p*		**0.001**		**0.001**		**0.04**		0.07	
Cohen’s d		**0.39**		**0.40**		**0.27**		0.23	
95% CI for Cohen’s d	Lower	0.13		0.14		0.01		−0.03	
	Upper	0.65		0.66		0.52		0.49	

Statistically significant results are written in boldface.

**Table 4:** Gender differences in physical activity during home confinement (A) and occupation disparities for alcohol consumption compared to before home confinement (B), *n* = 471.

**A**								
**Physical Activity Comparing to State before Isolation**	**Gender** ***N*, (%)**		**t**	**df**	***p***	**Cohen’s d**	**95% CI for Cohen’s d**	
	**Female**	**Male**					**Lower**	**Upper**
No exercising	123(30.5)	25(36.8)	−0.99	88.58	0.32	−0.13	−0.39	0.13
Started during isolation	70(17.4)	6(8.8)	2.17	111.13	0.033	0.25	−0.01	0.51
Less	72(17.9)	14(20.6)	−0.51	88.22	0.61	−0.07	−0.33	0.19
Same	71(17.6)	16(23.5)	−1.07	85.97	0.29	−0.15	−0.40	0.11
More	67(16.6)	7(10.3)	1.53	103.64	0.13	0.19	−0.07	0.44
**B**								
**Alcohol Consumption Comparing to State before Isolation**	**Occupation** ***N*, (%)**		**t**	**df**	***p***	**Cohen’s d**	**95% CI for Cohen’s d**	
	**Worker**	**Student**					**Lower**	**Upper**
**No alcohol**	**55(41.7)**	**178(52.5)**	**−2.13**	241.12	**0.034**	−**0.22**	−0.42	−0.16
Same	54(40.9)	128(37.8)	0.63	235.37	0.53	0.06	−0.14	0.27
More	23(17.4)	33(9.7)	2.09	196.10	**0.038**	**0.23**	0.02	0.43

Statistically significant results are written in boldface.

**Table 5:** Living alone (A) and new drug administration (B) during COVID-19 home confinement in terms of the Daily Life Fatigue scale (DLF), the Beck Depression Inventory (BDI), Revised UCLA Loneliness Scale (R-UCLA), and the Insomnia Severity Index (ISI) scales scores, *n* = 471.

**A**									
**Parameter**		**Are You Living Alone during COVID-19 Home Confinement?**							
		**DLF**		**BDI**		**R-UCLA**		**ISI**	
		**NO**	**YES**	**NO**	**YES**	**NO**	**YES**	**NO**	**YES**
Valid		414	57	414	57	414	57	414	57
Mean		7.33	9.11	13.82	16.63	41.36	47.10	15.74	16.07
Median		6.00	9.00	12.00	15.00	40.00	47.50	15.00	15.00
SD		5.81	6.38	10.49	11.48	14.18	11.28	6.90	7.35
Minimum		0.00	0.00	0.00	0.00	13.00	20.00	7.00	7.00
Maximum		24.00	23.00	50.00	54.00	63.00	74.00	35.00	32.00
t		−1.99		−1.75		−2.98		−0.32	
df		69.42		69.48		67.29		70.25	
*p*		**0.050**		0.08		**0.004**		0.75	
Cohen’s d		−**0.29**		−0.26		−**0.45**		−0.05	
95% CI for Cohen’s d	Lower	−0.57		−0.54		−0.73		−0.32	
	Upper	−0.01		0.03		−0.17		0.23	
**B**									
**Parameter**		**Are You Taking any New Drugs (Antidepressants, Anxiolytics, Sedatives, Other) during COVID-19 Home Confinement**							
		**DLF**		**BDI**		**R-UCLA**		**ISI**	
		**NO**	**YES**	**NO**	**YES**	**NO**	**YES**	**NO**	**YES**
Valid		425	46	425	46	425	46	425	46
Mean		7.17	10.98	13.38	21.67	41.34	48.64	15.31	20.17
Median		6.00	10.50	11.00	22.00	40.00	49.00	14.00	21.50
SD		5.77	6.12	10.51	8.88	11.69	10.61	6.71	7.63
Minimum		0.00	0.00	0.00	0.00	20.00	22.00	7.00	7.00
Maximum		24.00	24.00	54.00	39.00	74.00	70.00	34.00	35.00
t		−4.03		−5.93		−4.44		−4.15	
df		54.02		59.54		58.64		52.80	
*p*		**<0.001**		**<0.001**		**<0.001**		**<0.001**	
Cohen’s d		−**0.64**		−**0.86**		−**0.65**		−**0.68**	
95% CI for Cohen’s d	Lower	−0.97		−1.19		−0.98		−1.01	
	Upper	−0.31		−0.51		−0.33		−0.34	

Statistically significant results are written in boldface.
